# The Effects of Inhibiting Hedgehog Signaling Pathways by Using Specific Antagonist Cyclopamine on the Chondrogenic Differentiation of Mesenchymal Stem Cells

**DOI:** 10.3390/ijms14035966

**Published:** 2013-03-14

**Authors:** Xing Wu, Zheng-Dong Cai, Lei-Ming Lou, Zheng-Rong Chen

**Affiliations:** 1 Department of Orthopaedics, Shanghai tenth People’s Hospital, Tongji University School of Medicine, Shanghai 200072, China; E-Mails: Czd856@vip.sohu.com (Z.-D.C.); Shlml@hotmail.com (L.-M.L.); 2 Department of Orthopaedics, Shanghai Zhongshan Hospital, Fudan University School of Medicine, Shanghai 200032, China; E-Mail: doctorwx@hotmail.com

**Keywords:** Hedgehog, cyclopamine, mesenchymal stem cells, chondrocyte

## Abstract

This study aimed to investigate the effects of cyclopamine, a specific inhibitor of Hedgehog signaling pathways, on the chondrogenic differentiation of mesenchymal stem cells (MSCs). During culture, the experimental groups were treated with cyclopamine and their cell proliferation status was assessed using the MTT test. The extra-bone cellular matrix (ECM) and Collagen II (Col II) was detected by toluidine blue staining and immunohistochemistry of cells. The concentrations of Col II and aggrecan in the culture solution and cytosol were detected using ELISA on the 7th, 14th, and 21st days of cyclopamine induction. Gene and protein expression of Col II and aggrecan were analyzed on the 14th day of cyclopamine induction using real-time PCR and western blot analyses. No significant differences in proliferation of mesenchymal stem cells were found between the control group and the group treated with cyclopamine. Compared to the blank control group, the ECM level was low and the protein and mRNA concentrations of Collagen II (Col II) and aggrecan in the culture solution and cytosol, respectively, were significantly reduced in the experimental group. The Smo acted as a key point in the regulations of Hedgehog signaling pathway on the chondrogenic differentiation of rabbit MSCs.

## 1. Introduction

Hedgehog, a protein encoded by the segment polarity gene Hedgehog, plays an important role in embryonic development and organ formation. In vertebrates, almost all processes of embryonic development are regulated by Hedgehog. In recent years, much has been learned about Hedgehog and its signal transduction pathway, particularly concerning a novel and important function in bone and cartilage formation [1–3]. In higher vertebrates, the Hedgehog gene family contains at least three counterparts, referred to as Shh (Sonic hedgehog), Ihh (Indian hedgehog), and Dhh (Desert hedgehog). Studies have shown that Shh/Ihh is involved in the commitment, proliferation, and differentiation of cartilage progenitor cells, as well as in the differentiation of osteogenic cells, thus affecting the formation of osseous tissue [4–6].

Hedgehog cannot transcribe its signal without the help of Smoothened (Smo), a G protein-coupled multi-channel membrane protein. In the absence of Hh, Smo remains inactivated. However, when Hh is bound to Ptch, Smo performs its regulatory role and is activated to enhance the transcription activities of its target genes, which leads to the activation of a series of downstream signal molecules. Smo transcribes an intracellular signal to G1i (there are three homogenous Gli molecules in vertebrates, including Gli1, Gli2, and Gli3), a transcription factor that can enter the nucleus and trigger the expression of BMP, WNT, PTC, FGF, *etc.*[7]. Cyclopamine, an inhibitor of the Hh signaling pathway, is a plant steroidal alkaloid that inhibits Hh signaling mainly via inhibiting Smo [8]. The discovery of cyclopamine gave the study of the Hh signaling pathway greater clinical significance.

Collagen fibers contain three types of collagen: collagen I, collagen II, collagen III. Type II collagen (Col II) is a cartilage-specific collagen fiber that is secreted by chondroblasts, and it is a major component of the cartilage matrix framework. The expression of Col II is confined to cells with the chondrocyte phenotype, and therefore, the emergence of Col II may be a specific signal of chondrocyte differentiation, indicating increased synthesis of cartilage matrix [9,10]. Aggrecan is a structural protein found in a variety of extracellular matrices, and it is specific to the cartilage extracellular matrix, showing enhanced expression with chondrocyte maturation [11]. Therefore, in this study Col II and aggrecan were used as markers for differentiation of rabbit bone marrow MSCs into chondrocytes. Our previous studies have revealed, as many authors reported [12], the microgravity rotating culture system was more effective than routine standing 3D culture for the construction of tissue-engineered cartilage *in vitro*, particularly in views of formation of protein of cartilage matrix as Col II and Aggrecan.

Some research has shown [13] the effect of Shh on MSCs chondrogenesis; however the mechanisms of regulation of Hedgehog signal pathway are still unclear. We want to explore whether the Smo plays as a key point in the signal transduction of Hedgehog on MSCs chondrogenesis. In this study, we first constructed a chitosan cell carrier complex for rabbit MSCs, and then the cells were cultured in a conditional medium, which contained a chondrogenic inducer, by using a microgravity rotation model to generate tissue-engineered cartilage. Cyclopamine was added to the culture medium of the treatment group, and levels of protein and mRNA of Collagen II and aggrecan in the extracellular matrix and the cytosol, respectively, were measured in order to investigate how the Hedgehog signaling pathway affects the chondrogenic differentiation of mesenchymal stem cells.

## 2. Results

### 2.1. Morphological Observations and Toluidine Blue Staining of Cells

Cells were cultured on cove glass, and the medium was replaced every 48 h. Adherent cells showed uniform morphology with long fusiform spindles. After induction, cells were arranged in a spiral or spiral-like shape, and were transformed from long fusiform to polygonal and round, gradually becoming similar to chondrocytes. After 10 d to 13 d of induction, rabbit MSCs reached 80%–90% confluence. No significant differences were observed by inverted phase contrast microscope between the experimental group and the control group in growth rate or morphology.

**Figure 1:**
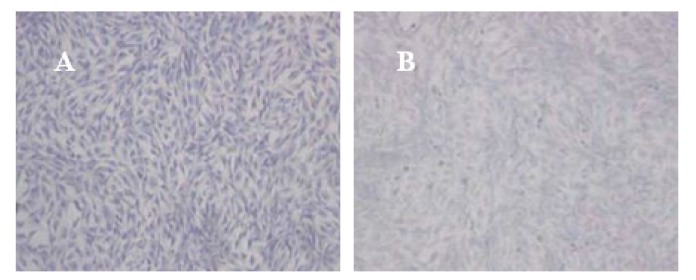
Toluidine blue staining for different rabbit MSCs cells clones (×4; **A**: the control group, **B**: the experiment group).

**Figure 2:**
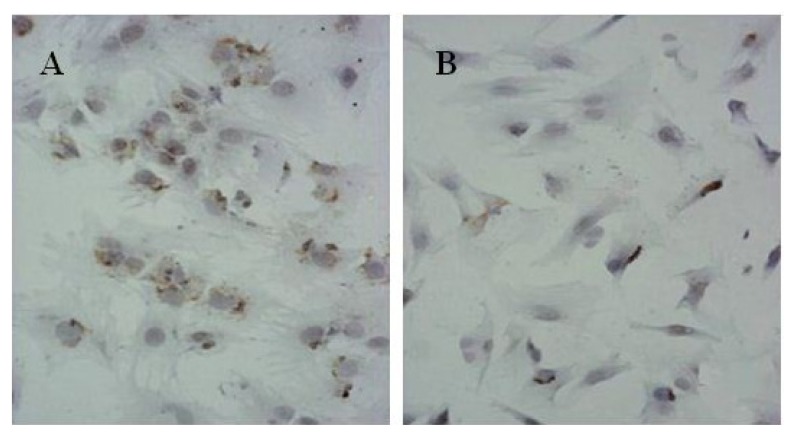
Collagen II expression using SP staining (×200; **A**: the control group, **B**: the experiment group).

The coloration of cells in the experiment group was darker than in the control group. The OD values of proteoglycans semi-determination were higher in the experiment group than in the control group (*p* < 0.01). These results indicated that the expressions of extra-bone cellular matrix proteins were relatively low in the experiment group (Table 1, Figure 1). The immunohistochemistry using SP staining showed the Col II expression in the control groups were obviously higher than the experiment group (Figure 2), which revealed the chondrogenesis of cells in the experiment were inhibited.

### 2.2. Cell Proliferation Viability Test

**Figure 3:**
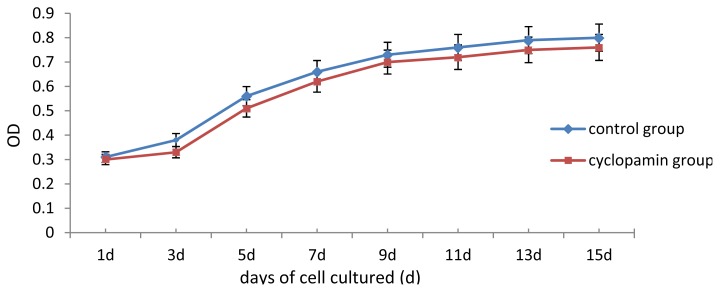
The cell proliferation curves of the cyclopamine treatment group and the blank control group by MTT test.

Rabbit MSCs cell proliferation increased gradually from 1 to 15 d after induction. After 10 d, the proliferation slowed. The results of the MTT test showed no significant difference between the cyclopamine treatment group and the blank control group in the cell proliferation curve (Figure 3).

### 2.3. ELISA

**Figure 4:**
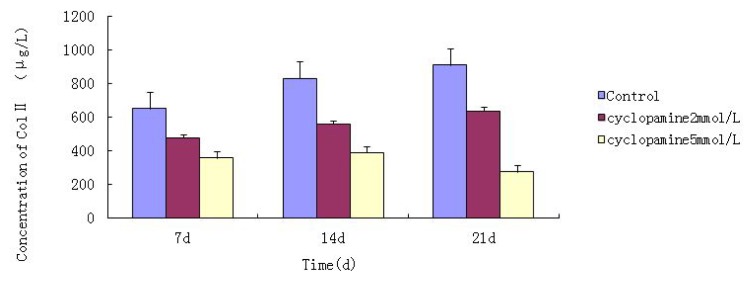
Comparison of the concentration of Col II in cells and the supernatant between the cyclopamine treatment group and the control group by ELISA.

**Figure 5:**
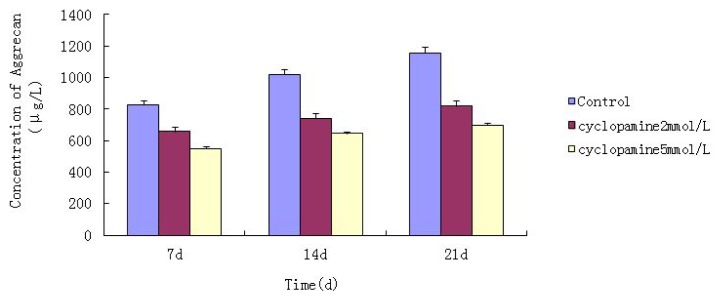
Comparison of the concentration of aggrecan in cells and the supernatant between the cyclopamine treatment group and the control group by ELISA.

An ELISA was conducted to measure total protein when cells were lysed at 7 d, 14 d and 21 d after treated with cyclopamine. The concentration of Col II and aggrecan in cells and the supernatant was significantly lower in the cyclopamine treatment group than in the control group (*p* < 0.05). In the 5 mmol/L group, the Col II and aggrecan concentrations decreased more obviously (*p* < 0.01) (Figures 4 and 5).

### 2.4. Analysis of mRNA Expression of Col II and Aggrecan Using Real Time RT-PCR

**Figure 6:**
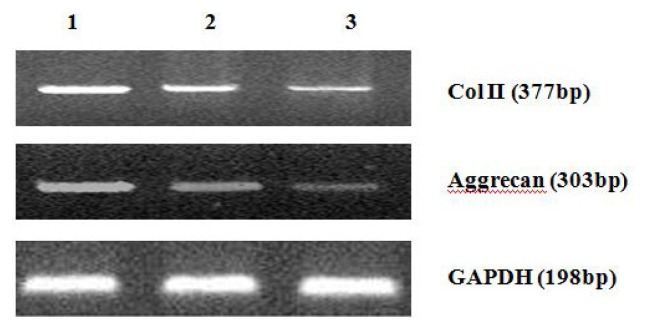
Detection of Col II, Aggrecan mRNA expression of cells by real time RT-PCR (1. the control group; 2. 2 mmol/L Cyclopamine group; 3. 5 mmol/L Cyclopamine group).

**Figure 7:**
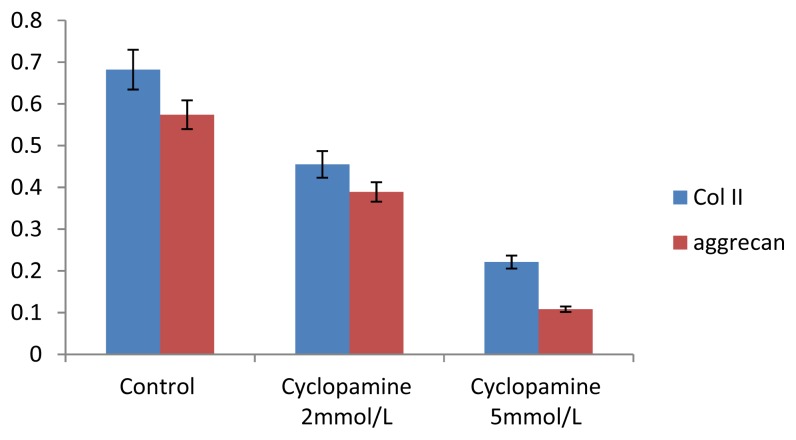
Analysis of the integrated optical density (IOD) of each patch by real-time PCR.

The gene amplification and melting curves for Col II and aggrecan illustrate that the target gene was effectively amplified and that the primer and probe used in the real-time PCR had good sensitivity to the target genes and no primer dimers were formed. The integrated optical density (IOD) of each patch was detected by an image-analysis system (AlphaImgaer 2000, San Leandro, CA, USA). The expression of Col II and aggrecan mRNA in cells in the cyclopamine treatment group was significantly lower than that in the control group (*p* < 0.05). In the 5 mmol/L group, Col II and aggrecan concentration decreased more obviously (*p* < 0.01) (Figures 6 and 7).

### 2.5. Western Blot Analysis of Protein Expression of Col II and Aggrecan

**Figure 8:**
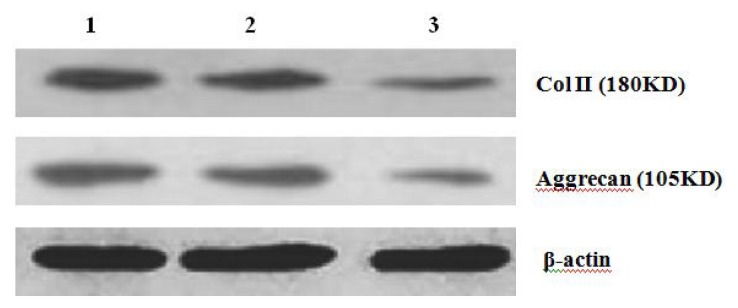
Detection of Col II, Aggrecan protein expression of cells by western blot (1. the control group; 2. 2 mmol/L Cyclopamine group; 3. 5 mmol/L Cyclopamine group).

**Figure 9:**
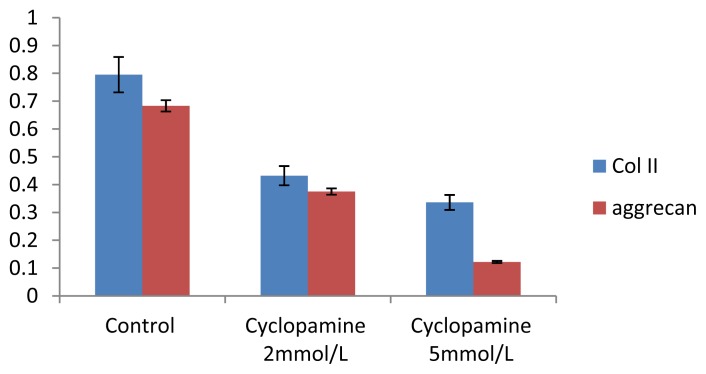
Analysis of the IOD of each patch by western blot.

The experiment was conducted at 7 d, 14 d, and 21 d after MSCs were treated with cyclopamine. After 2 weeks the concentrations of Col II and aggrecan in cells in the cyclopamine treatment group were significantly lower than that in the control group (*p* < 0.05). In the 5 mmol/L group, concentrations of Col II and aggrecan decreased more obviously (*p* < 0.01) (Figures 8 and 9).

## 3. Discussion

Studies have found that Hh signaling is involved in the regulation of bone growth and development. (1) Shh and development of osteoblast and chondrocyte. When chick embryo fibroblast cells overexpressing Shh were implanted intraperitoneally in athymic mice, the sites of heterotopic transplantation showed endochondral bone formation [14]. At the early phase of fracture repair, expression of Shh and Glil has been found in the periosteum and bone marrow cavity of the fracture site [15]. (2) Ihh and development of osteoblast and chondrocyte. Ihh can regulate growth plate chondrocyte proliferation and inhibit terminal differentiation; defects result in end limb deformities, accompanied by a decline in chondrocyte proliferation rate and expansion of the band of growth plate hypertrophic chondrocytes [16]. Recently some studies have showed [17] Hedgehog had a positive effect on the MSCs chondrogenesis, but few reports has been found regarding the key factors and targets regulated during this signal transduction process.

In our study, some external factors should be considered in case of interfering with the experimental results, so that the biases occur. One of our concerns was whether cyclopamine’s cytotoxicity could have inhibited cell growth and differentiation. Analysis of cell proliferation in this experiment found that cell proliferation of rabbit MSCs treated with 2 mmol/L, 5 mmol/L cyclopamine showed no significant difference from that in the blank control group. We, therefore, believe that the cytotoxicity of the drug at this concentration has no effect on the differentiation of MSCs. Another factor was the influence of fetal bovine serum (FBS) on the chondrogenesis of rabbit MSCs. 10% FBS has been reported to have promoting effect for MSCs chondrogenesis and been utilized as a component among chondrogenesis inducer [18].We tried to keep the uniform concentration of FBS applied to avoid the experimental bias. The third factor taken into considers was the cell cultured environments. In this study, the chitosan was used as a vector because it was similar to the cartilage ECM proteoglycans, to which MSCs easily attach [19,20]. In this study, we performed chondrocyte induction by complex of rabbit MSCs attaching into vector of chitosan *in vitro*. The MSCs attached to the 3D vector could transform to chondrocytes because of the sufficient cell-cell and cell-inducer contact because these complexes were obtained as the same cell pellet (CP) by centrifugation. Moreover we adopted the rotating cell culture system (RCCS) to imitate the microgravity environment in which cells, tissues, and culture solution could move in “free fall,” resulting in lower shear stress and more effective material transport [21,22]. To date literature is rare concerning the study of Hh signal regulation on MSCs chondrogenesis in the mimic microgravity environment. The uniformity of vector and culture system were monitored and kept. On the whole, sorts of negative factors and biases during MSCs chondrogenesis were averted to ensure the accuracy and objective of our study.

Further experiments showed that concentrations of Col II and aggrecan in the cells and supernatant of the test group were significantly reduced, and that expression levels of Col II and aggrecan mRNA and protein in cells as well as ECM level were also significantly reduced. These results of low levels of Col II and aggrecan indicated that inhibition of Hh signaling negatively affected chondrogenic differentiation from rabbit MSCs, and the Hh signaling pathway was involved in the regulation of the differentiation of MSCs into cartilage. Furthermore we deduced that the Smo molecular might play as a key factor in the signal transduction process, and Col II and aggrecan were found as the target genes up-regulated by Hh during rabbit MSCs chondrogenesis. These experimental results establish the basis for further study of the mechanism by which Hh regulates the differentiation of MSCs into cartilage. This project provided a novel direction in the study of the signal transduction pathway of chondrogenic differentiation of mesenchymal stem cells.

## 4. Experimental Section

### 4.1. Isolation, Culture and Identification of MSCs

Five milliliters (2 × 10^4^ U/L anticoagulant with heparin) of bone marrow was extracted via puncture of the intertrochanteric fossa of anesthetized New Zealand white rabbits. The bone marrow was then transferred into a centrifuge tube (PAA) preloaded with DMEM medium, centrifuged at room temperature, the fat and supernatant were removed, and the cells were resuspend in DMEM with 10% FBS and seeded in a culture flask with supplemented complete medium (10% FBS, Penicillin G 10^5^ U/L, streptomycin 100 mg/L, DMEM Low Glucose), and kept at 37 °C in a humidified atmosphere containing 5% CO_2_. Nonadherent cells were removed during the first medium replacement after 3 days in culture, and the medium was replaced every 3 days. MSCs were identified using technology of flow cytometer in according to expression of cell phenotype as CD29+, CD44+, CD71+, CD105+, CD90+ and CD14−, CD34−, CD45−, CD11b−.

### 4.2. Preparation of Cells and PGA Carrier

The PGA (Equl Company, Shanghai, China) was sterilized by soaking a piece approximately 4 mm × 4 mm × 4 mm in 100 mL of isopropanol for 30 min, then washed with sterile distilled water. After sterilization, the PGA was soaked in DMEM with 10% FBS for 24 h. The second generation cells were digested with 0.25% trypsin. Thereafter, 1 × 10^6^ cells were seeded in PGA and differentiation was induced.

### 4.3. Chondrogenic Differentiation *in Vitro* of MSCs and Test Groups

The carrier/MSCs complex was transferred into 50-mL HARV (Synthecon Inc., Houston, TX, USA) containers. Induced medium (10% FBS + H-DMEM, TGF-β 110 ng/mL, Vc 50 μg/mL, Dex 10^−7^ mol/L, insulin 5 μg/mL) was added to the blank control group. Cyclopamine at concentrations of 2 mmol/L and 5 mmol/L was added to the induced medium of the test groups. Bubbles were evacuated from the containers, which were then fixed in the rotary cell culture system, and incubated at 37 °C in a humidified atmosphere containing 5% CO_2_. The rotation speed was 20 rpm, and the medium was replaced every 2 days.

### 4.4. MTT Test for the Proliferation of MSCs

Two carriers were removed from the control and cyclopamine 5 mmol/L groups at the same point in time 1 d, 3 d, 5 d, 7 d, 9 d, 11 d, 13 d and 15 d after the cells were seeded. Each carrier was incubated with CCK-8 solution for 2 h, and then the MTT test was conducted. The absorbance value (450 nm) was read using a microplate reader, with a reference of 600 nm. Absorbance values were averaged for drawing the cell proliferation curve. 3 replicates were performed for each time point and group.

### 4.5. Proteoglycans Semi-Determination by Toluidine Blue Staining after Induction

Complexes of vector and cells from various groups were collected in 12-well plates. The vector precipitate was dissolved by solvent and the cell suspension was collected. A coverslip was placed in the cell suspension and the cell creep plates were generated. The creep plates were removed, washed twice with NS, and stationary liquid (0.3 M NaCl + 70% ethyl alcohol) was added to the plates. The plates were reacted at room temperature for 1 h, adsorbed drying, and placed on a glass slide. The slides were washed twice with PBS for 5 min and adsorbed drying. Toluidine blue (2%) stain was added for 4 h. Ethyl alcohol (95%) was subsequently added and the excess dye was washed out and the slides were fixed by neutral balsam. The degree of toluidine blue staining was observed by optical microscope. The Northing medical image analysis system was applied to determine the average optical density (OD) of positive staining in each visual fields. Five visual fields were taken randomly in each sample, and OD was denoted as *χ̄ ± s*.

### 4.6. Immunohistochemistry

10^4^ cells were collected in 6 wells plates. A coverslip coated with polylysine was placed at the bottom of the plate. Cells were conventional cultured in complete medium with 10% FBS. Immunohistochemistry with SP technology were applied for detection of Collagen II (Col II) protein expression according to the manual instructions of product kit. The benzdine diaminodiphenyl(DAB) for chromogenic substrate and the hematoxylin for contrast staining were utilized. The reagents without monoclonal antibodies of Col II were used for negative control.

### 4.7. Detection of the Concentration of Col II and Aggrecan in Cells and Medium by Using ELISA

Cells from the treatment group receiving 5 mmol/L cyclopamine were digested and collected at 7 d, 14 d, and 21 d after being induced. Cells were diluted and suspended with PBS (pH 7.2–7.4).The concentration of the cells was 10^6^/mL. The cells were lysed to release their intracellular components by adding tissue protein extraction reagent. They were then centrifuged for 20 min at 2000–3000 rpm and the supernatant was collected. If precipitate formed, they were centrifuged again. Every group was transferred to 2 wells, each experiment was repeated 3 times, and each sample set was transferred to 3 wells. Operating in accordance with the manufacturer’s instructions, a standard curve was drawn to calculate the corresponding concentration, and a chart was plotted with the concentrations at the vertical axis and the different treatment groups at different times of the day as the abscissa.

### 4.8. Analysis of mRNA Expression of Col II and Aggrecan Using Real-Time PCR

After 2 weeks of chondrogenic differentiation, induction was aborted. Samples of PGA/MSCs cell carrier complex were taken from the treatment group and the control group, and the medium was removed from the carrier. Carriers were washed twice with PBS. PGA was dissolved using PGA solvent in order to suspend the cells. Total RNA was extracted using Trizol. Referring to the cDNA sequences in GenBank, primers for the target genes (PAGE purification) were synthesized by Shanghai Biocolor BioScience & Technology Company. The sequences of the primers used were as follows:

1. Col II: sense 5′-CCAGGTCAAGATGGTC-3′,
  antisense 5′-CTTCAGCACCTGTCTCACCA-3′;
2. aggrecan: sense 5′-CACTGTTACCGCCACTTCCC-3′,
  antisense 5′-GACATCGTTCCACTCGCCCT-3′;
3. GAPDH, which was used as a reference gene:
  sense 5′-TGGAAATCCCATCACCATCT-3′,
  antisense 5′-GTTCATGCCCATCACAAACA-3′.

One microgram of total mRNA was mixed with 1 μL of the 3′-primer, and then incubated for 10 min at 70 °C. We synthesized cDNA by using Exscript™ RT reagent Kit (TaKaRa, Otsu, Japan), following the manufacturer’s instructions. One microgram of cDNA was used for real-time PCR analysis. The PCR was conducted in a 50-μL reaction volume by using a 48-well MiniOpticon™ System (Bio-Rad, Philadelphia, PA, USA) reaction system. The cDNA was predenatured at 95 °C for 10 s, followed by, 45 cycles of amplification, each cycle consisting of denaturation at 95 °C for 6 s and annealing at 62 °C for 20 s. Thereafter, a melting curve was plotted. Specificity of the RT-PCR amplification products was identified through a 1% agarose gel electrophoresis and analysis of the melting curve for each gene. A series of 4-fold dilutions of the cDNA template were used to detect RT-PCR sensitivity and to build a standard curve. All samples were then normalized, using the reference gene as a standard (correcting for the initial amount of RNA). To determine the relative expression level of each target gene in the different samples, each test sample was divided into 3 and the experiment was repeated at least 3 times.

### 4.9. Western Blot Analysis of Expression of Col II and Aggrecan

After 2 weeks of chondrogenic differentiation, induction was aborted. Samples of PGA/MSCs cell carrier complex were taken from the treatment group and the control group, and the medium was removed from the carrier. Carriers were washed twice with PBS. Cells were lysed using M-PER protein lysate at 4 °C, and centrifuged at 1500 × *g* for 15 min. The supernatant was transferred into a new EP tube, and after protein quantification using SDS-PAGE electrophoresis according to conventional methods, the separated proteins were transferred to nitrocellulose membranes and incubated with blocking solution overnight. The proteins were then incubated with anti-collagen II and aggrecan primary antibody overnight and then rinsed thoroughly with TBS. A second antibody was added to the reaction mixture and they were incubated at 37 °C for 1 h; thereafter, the mixture was stained using the SP method. Three replicates were performed.

### 4.10. Statistical Analysis

Statistical analyses were conducted using SPSS 10.0 software. The results are expressed as mean ± standard deviation. Comparisons of the experimental group, the control group, and the blank group were conducted using the Kruskal-Wallis test.

## 5. Conclusions

Our data showed that no cytotoxicity of the drug on the differentiation of rabbit MSCs had been found when they were treated with a test concentration of 2 mmol/L~5 mmol/L cyclopamine. Further experiments showed that compared with blank control, the concentrations of Col II and aggrecan in the cells and supernatant of the test group as well as ECM level were significantly reduced, and that expression levels of Col II and aggrecan mRNA and protein in cells were also significantly reduced. These results simultaneously indicated that the Hh-Smo signaling pathway was involved in the regulation of the differentiation of rabbit MSCs into cartilage, and the Smo played an important role as a capital and key factor; moreover Col II and aggrecan were found as the target genes up-regulated by Hh during rabbit MSCs chondrogenesis.

## Figures and Tables

**Table 1:** Proteoglycans semi-determination by toluidine blue staining.

Groups	OD values
Controgroup	0.882 ± 0.052
Experiment l group	0.365 ± 0.028
*p-*Value (*t* test) *	<0.01

* : OD values in the experiment group compared with the control group.
